# A common ground: an in silico assessment of the sources of intrinsic ex vivo resistance to venetoclax in acute myeloid leukemia

**DOI:** 10.1016/j.htct.2025.103758

**Published:** 2025-04-12

**Authors:** Brunno Gilberto Santos de Macedo, Manuela Albuquerque de Melo, Diego Antonio Pereira-Martins, João Agostinho Machado-Neto, Fabiola Traina

**Affiliations:** a Department of Medical Images, Hematology, and Oncology, University of São Paulo at Ribeirão Preto Medical School, Ribeirão Preto, São Paulo, Brazil; b Department of Hematology, University Medical Center Groningen, Groningen, The Netherlands; c Department of Pharmacology, University of São Paulo, São Paulo, São Paulo, Brazil

**Keywords:** Acute myeloid leukemia, Drug resistance, Targeted molecular therapies, Venetoclax

## Abstract

Venetoclax is a promising alternative for patients with acute myeloid leukemia who are considered unfit for conventional chemotherapy; however, its employment still faces challenges mostly related to drug resistance. Here, we provide further biological mechanisms underlying the previously described and potentially novel intrinsic sources of poor response to venetoclax departing from *ex vivo* response data. Acute myeloid leukemia data including *FLT3* mutation status, gene expression data, and *ex vivo* response data were extracted from the publicly available BeatAML 1.0 study database and aided sample categorization that supported differential gene expression analysis that, in turn, supported gene set enrichment analysis. CIBERSORTx-based bulk RNA sequencing deconvolution of BeatAML 1.0 data allowed us to categorize samples according to their cell type content. We observed that inflammation-related gene sets, such as cytokines and inflammatory response, NLRP3 inflammasome activation, and activation of adaptive immune response, were concordantly positively enriched across all the conditions reported to be associated with poor *ex vivo* venetoclax response, whereas samples from good *ex vivo* responders’ mostly enriched gene sets related to mitochondrial activity, and early myeloid progenitor cell molecular programs. Besides the alternative reliance on *BCL2A1*, we highlight inflammation as a common element present across multiple sources of venetoclax *ex vivo* response modulation in acute myeloid leukemia samples. Hence, a potential key modulator for venetoclax response.

## Introduction

The growing understanding of the molecular diversity and pathogenesis of acute myeloid leukemia (AML) has not only contributed significantly to defining prognosis and guiding clinical decisions,^1^^,^^2^ but has also opened up therapeutic avenues. These include opportunities for targeted treatments based on molecular profiles and, notably, the development of more tolerable strategies through the use of small molecule inhibitors.^2^, ^3^, ^4^ The most promising and notable examples of Food and Drug Administration (FDA)-approved molecularly-oriented targeted approaches comprise FLT3, IDH1/2, and BCL-2 inhibition.^2^, ^3^, ^4^, ^5^, ^6^

In this context, the BCL-2 inhibitor venetoclax has drawn significant attention from the scientific community as a small molecule targeted therapy for AML. This strategy represents a promising alternative for patients who may not qualify for standard intensive induction chemotherapy regimens. Venetoclax is believed to target leukemic stem cells (LSCs) and their metabolic characteristics, which contribute to long-lasting remission.^7^, ^8^, ^9^, ^10^

However, some of the significant mechanisms of resistance to venetoclax are mostly related to energy metabolism plasticity, and, most importantly, to the attenuation of *BCL2* dependency for survival.^6^^,^^9^^,^^11^ This can be observed upon BCL2 downregulation, upregulation of additional BH3-family anti-apoptotic proteins such as BCL-XL, BCL-W, and MCL1, and even monocytic-like AML populations were observed to be inherently resistant and positively selected upon treatment with venetoclax.^2^^,^^5^^,^^6^^,^^9^^,^^11^^,^^12^ Activation of other underlying parallel survival alterations such as gain of *FLT3*-ITD or *TP53* loss-of-function mutations are also reported as additional primary sources of resistance to venetoclax.^6^ Another recently described mechanism of resistance to venetoclax encompasses the nicotinamide metabolism, prominently orchestrated by the enzyme nicotinamide phosphoribosyl transferase encoded by the *NAMPT* gene, in relapsed and refractory AML LSCs.^13^

Venetoclax exhibits restricted efficacy in relapsed or refractory AML, primarily owing to the presence of specific mechanisms.^9^ Relapsed cells often depend on anti-apoptotic proteins other than BCL-2 or demonstrate increased metabolic adaptability to compensate for the disruption caused by BCL-2 inhibition. For example, they may increase fatty acid intake and activate alternative metabolic pathways such as mitochondrial fatty acid beta-oxidation to fuel the tricarboxylic acid cycle (TCA) and generate adenosine triphosphate.^9^

In the light of the diversity of these mechanisms, this study sought to unravel additional biological mechanisms underlying already described and novel sources of intrinsic and acquired poor response to venetoclax from an *ex vivo* screening perspective.

## Methods

### Data acquisition

Mutation status, gene expression (RNA sequencing), and *ex vivo* response data from primary AML bone marrow mononuclear cell samples were obtained from the BeatAML 1.0 functional genomic study cohort through the supplementary documentation in Tyner et al.^14^, cBioPortal (cbioportal.org) repository, and the BeatAML 1.0 associated data viewer, Vizome (vizome.org). Along with transcriptional deconvolution data, the aforementioned data aided sample categorization for the following methods and statistical processing (Supplemental Figure 1).

### Gene set enrichment analysis

The gene set enrichment analysis (RRID:SCR_003199) was performed from the gene expression logarithm fold change (logFC)-based genes pre-ranking. LogFC values were obtained from differential gene expression analyses using the *edgeR* (RRID:SCR_012802) and *limma* (RRID:SCR_010943) Bioconductor R packages under the *limma*-*voom* algorithm. The LogFC output of BH3 family genes –*BAD, BAK1, BAX, BBC3, BCL2, BCL2A1, BCL2L1, BCL2L11, BCL-W, BIM, BID, BOK*, and *PMAIP1* –was graphically represented through a heatmap built employing the *ComplexHeatmap* (RRID:SCR_017270) Bioconductor R package and clustered according to the Euclidean distance.

The pre-ranked genes also served as the input for the *fgsea* (RRID:SCR_020938) Bioconductor R package. The gene sets are obtainable from the Molecular Signature Database (MSigDB - gsea-msigdb.org).^15^ The pre-ranked genes enrichment was submitted to 10,000 permutations under weighted enrichment statistics. The level of significance was pre-established at 5 % and adjusted to a false discovery rate of 25 %. This work employed the Gene Ontology: Biological Process (GOBP; 7751 gene sets) and the curated (C2; 7233 gene sets) collections of human gene sets that were loaded using the *qusage* Bioconductor R package. The gene sets were selected by convenience under the statistical significance and false discovery rate criteria and graphically represented using the *ComplexHeatmap* (RRID:SCR_017270) Bioconductor R 4.3.1 package.

### Deconvolution analysis

The BeatAML 1.0 cohort's bulk RNAseq gene expression data was submitted to the CIBERSORTx tool (https://cibersortx.stanford.edu/).^16^ The CIBERSORTx tool, in the current context, promoted bone marrow mononuclear cell gene expression signal deconvolution into the different cell populations that compose it and assigned a compartmental score to each type of cell population according to a reference to single-cell RNA sequencing data, in this case van Galen et al. (leukemia and primary healthy scRNAseq bone marrow samples).^17^ The compartment score indicates which cell type is predominant within a sample, therefore its cellular composition regarding abundance; as an indirect measurement of how much of a particular cell type contributes to the total average gene expression signal. CIBERSORTx-derived deconvolution data from the BeatAML 1.0 cohort is available in the supplementary material of Zeng et al.^18^

### Statistical analyses

All statistical analyses were performed using R programming language version 4.3.1 (R Core Team (2022) - https://www.R-project.org/) (RRID:SCR_001905) and RStudio Integrated Development Environment (IDE) version 2023.03.0 + 386 (RStudio Team (2020) –http://www.rstudio.com/) (RRID:SCR_000432). The established level of significance (α) was 5 % for all the analyses. Contingency tables were analyzed using Fisher's exact test and the effect size was measured by the odds ratio (OR) and a 95 % confidence interval.

## Results

Inflammation-related and mature blood cell-related gene sets are consistently enriched across the conditions associated with poor intrinsic venetoclax *ex vivo* response.

**Figure 1:**
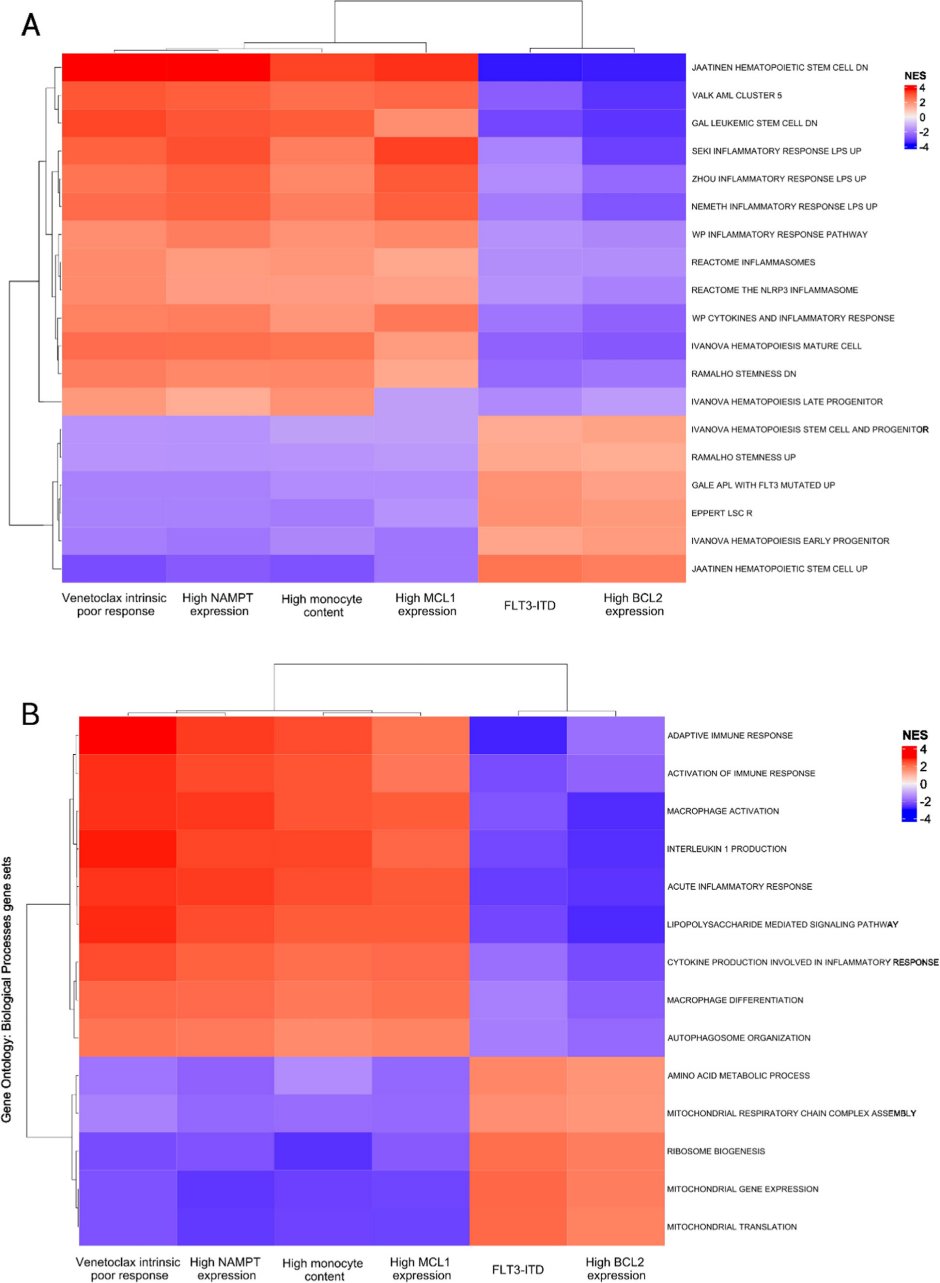
Biological processes profiling regarding venetoclax response modulators according to gene set enrichment analysis In the columns, different conditions associated with intrinsic venetoclax response modulation as *NAMPT, MCL1*, and *BCL2* gene expression levels, AML samples monocytic cell content, the presence of the *FLT3-*ITD mutation, alongside intrinsic venetoclax *ex vivo* response itself. In the rows, different gene sets from the curated human gene sets collection (A) and the Gene Ontology: Biological Processes collection (B). Each heatmap cell represents a normalized enrichment score (NES) value. Darker shades of red represent higher NES values, which means that this particular biological process or entity is related to the condition of interest. On the other hand, darker shades of blue stand for lower NES values, which means that a specific biological process or entity is more related to the condition of interest counterpart.

In both tested human gene set collections, conditions such as higher *NAMPT* and *MCL1* expression dichotomized by gene expression median, along with higher sample monocyte content, dichotomized according to CIBERSORTx score median value, displayed similarities to the intrinsic venetoclax poor response reference molecular signature (Figure 1). Conversely, the *FLT3*-ITD mutation and higher *BCL2* gene expression were molecular features associated with good *ex vivo* response to venetoclax (Figure 1). Inflammation-related biological processes such as inflammasome activation and cytokine production, macrophage activation and mature hematopoietic cells were consistently present in the molecular signature compatible with poor intrinsic *ex vivo* response to venetoclax (Figure 1). In contrast to this observation, gene sets related to mitochondrial activity, amino acid metabolism, and immature hematopoietic cells, including hematopoietic and LSCs, were found to be enriched within the molecular signature compatible with venetoclax sensitivity (Figure 1).

Acute myeloid leukemia samples with higher *MCL1* and *NAMPT* gene expressions, and higher monocytic cell content present increased likelihood of poor intrinsic *ex vivo* response to venetoclax.

**Figure 2:**
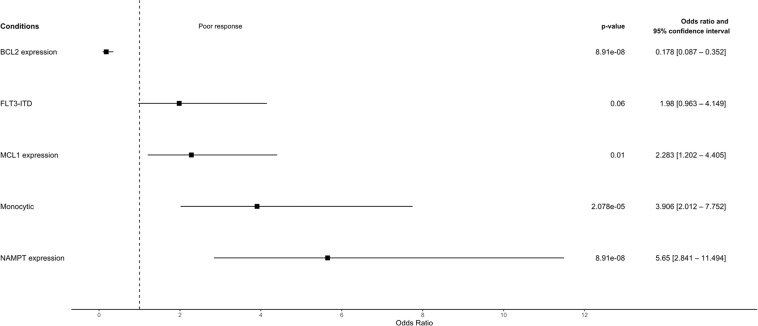
The extent of association between Venetoclax *ex vivo* response and its reported sources of intrinsic resistance This forest plot displays the odds ratio and 95 % confidence interval (CI) for venetoclax *ex vivo* response across multiple reported sources of venetoclax response modulators, either promoting resistance or sensitivity. Each horizontal line represents a condition: ‘gene expression’ groups have the ‘higher expression’ group as reference; the *FLT3-*ITD mutation presence as reference; and monocytic content, higher monocytic signature as reference according to CIBERSORTx analysis of the samples. The OR and 95 % CI of each association are respectively represented as the square and the horizontal line on the plot. The vertical line intercepts the x-axis at an OR of 1 for which no association is observed.

The association analysis (Figure 2) presented higher *BCL2* expression as a factor which is strongly associated with good intrinsic *ex vivo* response to venetoclax (OR: 0.178; 95 % CI: 0.087–0.352). On the other hand, a higher *MCL1* expression made the samples over twice as likely to present poor intrinsic *ex vivo* response to venetoclax (OR: 2.28; 95 % CI: 1.202–4.405), a higher monocytic signature increases the likelihood by almost four times (OR: 3.906; 95 % CI: 2.012–7.752], and higher levels of *NAMPT* expression were associated with a poor likelihood of intrinsic *ex vivo* response to venetoclax (OR: 5.65; 95 % CI: 2.841–11.494). These results denote that our analyses are concordant with the reported venetoclax response modulation in current literature while offering an *ex vivo* perspective and a mathematical standpoint for the likelihood of intrinsic response.

BCL2A1 is a key player in differentially expressed BH3 family genes associated with poor intrinsic *ex vivo* response to venetoclax

**Figure 3:**
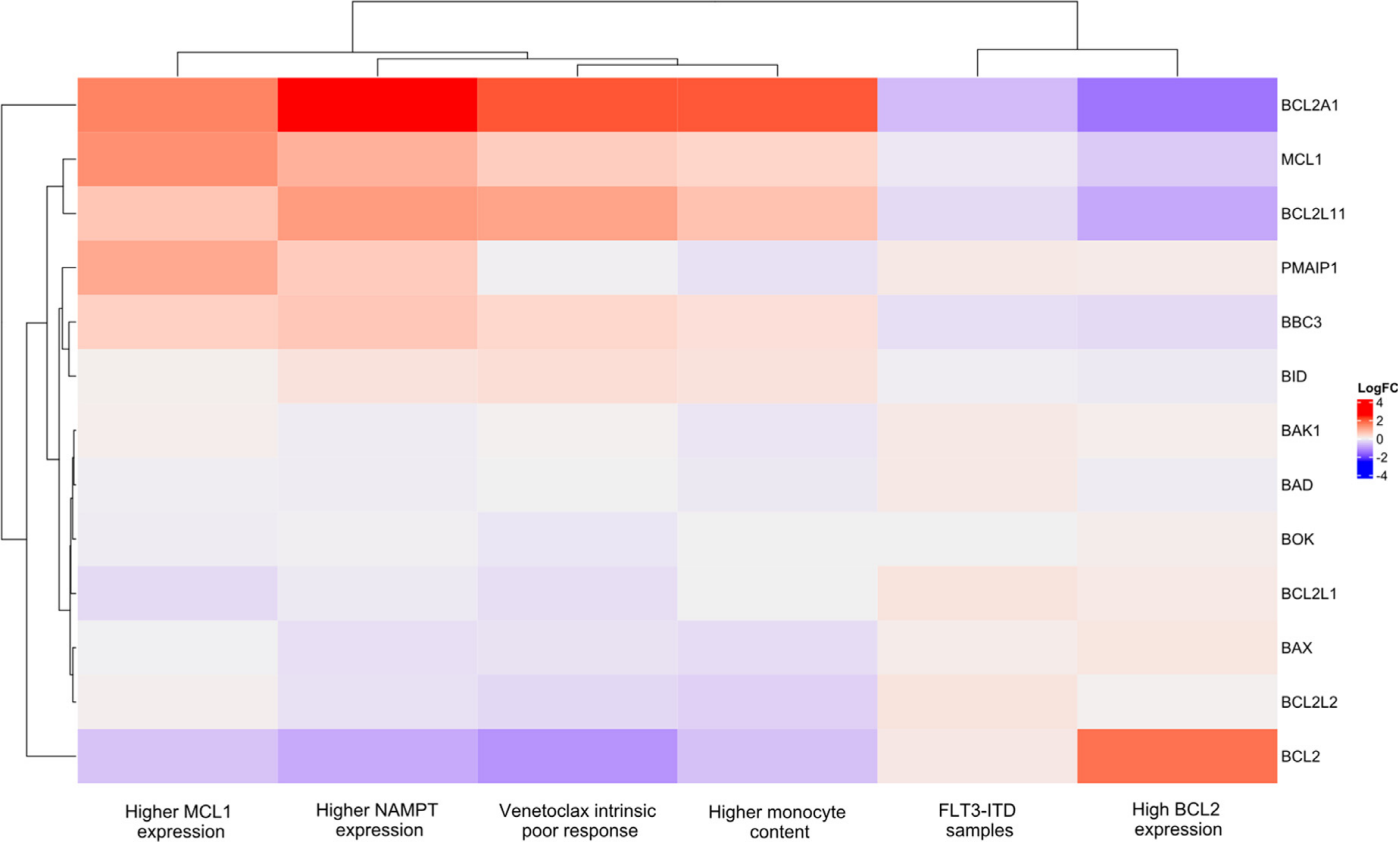
Heatmap of the logarithm fold-change (LogFC) values on comparing modulators of venetoclax response. In the columns, BH3 family genes including apoptosis suppressors (*BCL2, BCL2A1, BCL2L1* or *BCL-XL*, and *BCL-W*), and activators (*BAD, BAK1, BAX, BBC3* a.k.a. *PUMA, BCL2L11* a.k.a. *BIM, BID, BOK*, and *PMAIP1* a.k.a. *NOXA*). In the rows, venetoclax response reported modulators as BCL2, MCL1, and NAMPT expression having higher expression as reference group for differential gene expression comparisons; sample monocytic content having higher monocytic content as reference group; *FLT3-*ITD mutation status having *FLT3-*ITD as reference group; and intrinsic *ex vivo* response to venetoclax having poor response as reference group. Each cell of the heatmap displays a LogFC value; the cells in shades of red represent upregulated genes in the reference groups, whereas the cells in shades of blue represent downregulated genes in the reference groups.

The heatmap of differentially expressed genes (Figure 3) revealed a molecular signature for poor response strongly based on the *BCL2A1* and *BCL2* opposite gene expression behaviors in this set of samples. Conditions associated with poor response significantly upregulated *BCL2A1* while downregulated *BCL2*. Conversely, *FLT3-*ITD mutated samples were clustered along a condition widely described as a good response signature based mainly on *BCL2A1* downregulation.

## Discussion

Due to its performance in early phases of clinical trials, in 2018, venetoclax had its approval by the FDA accelerated as long as it was combined with hypomethylating agents or low dose of cytarabine for AML patients who were over 75 years old and presenting comorbidities that forbid intensive chemotherapy.^3^^,^^6^^,^^19^ The regular approval of venetoclax for newly-diagnosed untreated AML patients was granted by the FDA only 2 years later.

Considering its relatively brief regular approval time, it is important to further characterize and report sources of resistance and potential obstacles its employment might face. Assessing primary sources of resistance would provide a better decision-making capability while expanding the knowledge on mechanisms of drug resistance, how to address these mechanisms in order to circumvent these events, and expand the benefits of the drug.

In the BeatAML 1.0 cohort, we found that higher *NAMPT* and *MCL1* expressions and higher monocytic cell content were associated with poorer intrinsic response to venetoclax. These findings are consonant with the current literature.^12^^,^^13^^,^^20^ In fact, these findings even complement observations in the current literature regarding *NAMPT*-mediated venetoclax resistance, not only making the gene a source of acquired resistance present in relapsed and refractory AML LSCs,^13^ but also a prominent source of intrinsic *ex vivo* resistance to venetoclax.

In agreement with and complementing the findings of Zhao et al., we also observed that samples that enriched monocytes in their cellular composition were inherently resistant to venetoclax.^21^ Although our data were not classified according to the French-American-British morphological classification, our gene set enrichment analysis revealed the presence of a gene set based on cluster 5 of AML samples of Valk et al., which are morphologically classified as myelomonocytic and monocytic leukemia.^22^

Waclawiczek et al. described that the current widely available AML therapy often spares cellular populations capable of evading it and driving relapse.^2^ Our findings regarding cell population composition within studied samples and their response to venetoclax have shown that monocyte cells are enriched in samples from poor responders. In contrast, hematopoietic stem cell-like AML cells and early myeloid progenitors were enriched in good responders revealing two cell populations that are not substantially targeted by conventional chemotherapy but that present opposite sensitivity behaviors.

The enrichment of molecular signatures related to mitochondrial respiration and amino acid metabolism, along with the molecular signature compatible with early hematopoietic progenitors and LSCs, corroborates the metabolic behavior described as associated with de novo AML LSCs that are responsive to venetoclax.^9^^,^^23^ The combination of venetoclax and azacytidine was described as inhibiting amino acid metabolism and impairing oxidative phosphorylation in LSC.^9^^,^^23^

Finding inflammation-related gene sets to be homogeneously enriched across different conditions reported as modulators of venetoclax response not only provides a potential biological process that consistently underlies venetoclax resistance across multiple conditions reported to promote intrinsic refractoriness, but also highlights a process currently poorly described as a basis for venetoclax resistance.

Currently, a single work directly states that inflammation is a promoter of venetoclax resistance in line with our findings. Wang et al. described that interferon-gamma (IFNγ) signaling was strongly correlated with venetoclax resistance, and treating primary AML cells with IFNγ increased their resistance to venetoclax, suggesting that IFNγ inhibition may be a potential strategy to bypass venetoclax resistance.^24^

It is also noteworthy that the patients considered ineligible for conventional chemotherapy that would benefit from venetoclax treatment are often under a tendency to maintain an increased chronic basal inflammatory state, mostly caused by age-related telomere shortening and associated comorbidities, that could, according to our data, mitigate the drug's efficacy.

This work also describes, for the first time, the effects of inflammasome activation over venetoclax response. The inflammasome was previously described as an enhancer of the fitness of AML cells,^25^ and its main byproducts were credited to provide a beneficial microenvironment for the selection of leukemia cells at the expense of healthy hematopoietic cells.^25^^,^^26^ We emphasize the activation process as a source of intrinsic venetoclax resistance.

Finally, our findings regarding the presence of *FLT3*-ITD were consistently associated with increased venetoclax sensitivity in contrast to those described by Liu et al.^6^ Even though we failed to establish a likely association between the presence of *FLT3*-ITD and poor intrinsic *ex vivo* venetoclax response, the GSEA-based molecular signature of *FLT3*-ITD was not only constantly similar to *BCL2* upregulation, a biologically supported condition for good response to venetoclax, but also opposed to the molecular signatures related to venetoclax resistance.

Another piece of evidence that substantiates *FLT3*-ITD as a molecular entity linked to sensitivity to venetoclax is that *FLT3*-ITD samples presented a BH3 protein family differential gene expression profile very similar to *BCL2* high expression samples. Indeed, we observed that poor responders to venetoclax coordinately upregulated *BCL2A1* as an alternative antiapoptotic BH3 family protein to rely on. Differing from the observations of Pei et al., the monocytic population studied here was seemingly more reliant on the BCL2A1 protein instead of the MCL1 protein.^12^ *BCL2A1* was also shown to increase venetoclax inhibitory concentration (IC_50_) to 20-fold in AML cell models transduced with a lentivirus containing doxycycline-induced *BCL2A1.*^20^

## Conclusion

Taken together, this work offers a much-needed common ground across multiple response factors of venetoclax-based therapy. It highlights inflammation and, for the first time, identifies inflammasome activation as a potentially crucial biological process in venetoclax response, alongside sample cellular composition and developmental stage. This work also further solidifies *BCL2A1* as a relevant target to address venetoclax resistance, and emphasizes the importance of energy metabolism on the intrinsic venetoclax response. We acknowledge that a primary limitation of this work lies in the nature of the employed data, which is based on an *ex vivo* screening.

## Conflicts of interest

The authors declare no potential conflicts of interest.

## Appendix Supplementary materials

Image, application 1
