# Supplementary material for: A common ground: an in silico assessment of the sources of intrinsic ex vivo resistance to venetoclax in acute myeloid leukemia
Source: Hematol Transfus Cell Ther. 2025 Apr 12;47(2):103758. doi: 10.1016/j.htct.2025.103758 (PMC12019820; doi:10.1016/j.htct.2025.103758)
Supplement: Supplementary file 1 [file mmc1.pdf]

Supplemental Figure 1

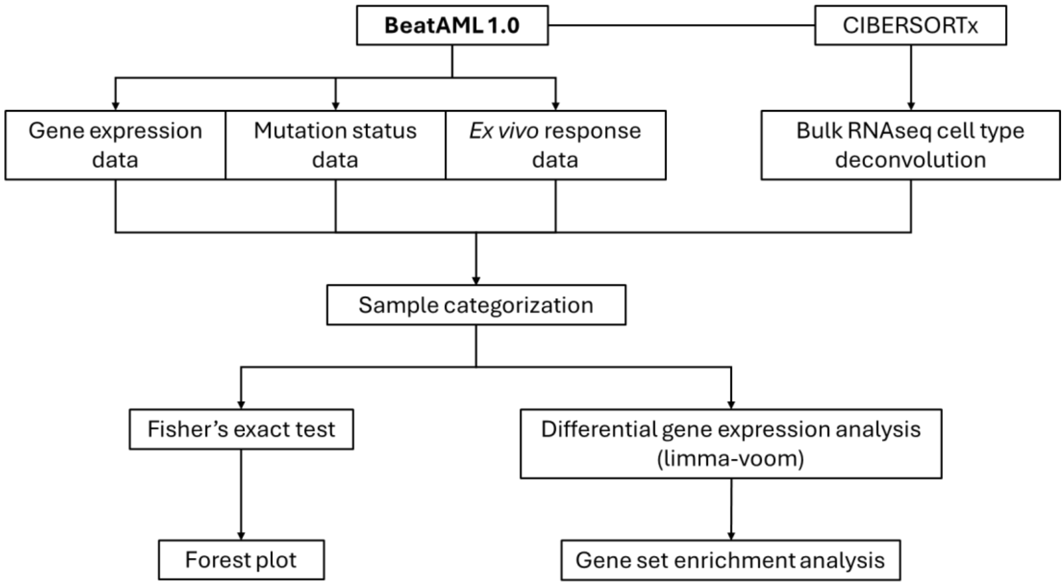

Supplemental Figure 1. Gene expression, mutation status, and *ex vivo* response data were acquired from the BeatAML 1.0 functional genomic study data viewer (vizome.org) and supplemental data on the original publication for the dataset (https://doi.org/10.1038/s41586-018-0623-z). Parallelly, CIBERSORTx-based bulk RNA sequencing data deconvolution analysis was performed and made publicly available (https://doi.org/10.1038/s41587-019-0114-2). Altogether, the cohort’s data was employed in sample categorization based on the median for continuous variables as *ex vivo* response, gene expression data and deconvolution data, respectively measured in area under the curve (AUC), counts per million, and score of transcriptomic contribution. The categories supported further analyses as differential gene expression under limma-voom algorithm which entailed gene set enrichment analysis; and Fisher’s exact test for association between sample measurable features and their phenotypic traits.
